# Evaluation of inner retinal layers as biomarkers in mild cognitive impairment to moderate Alzheimer’s disease

**DOI:** 10.1371/journal.pone.0192646

**Published:** 2018-02-08

**Authors:** Eleonora M. Lad, Dibyendu Mukherjee, Sandra S. Stinnett, Scott W. Cousins, Guy G. Potter, James R. Burke, Sina Farsiu, Heather E. Whitson

**Affiliations:** 1 Department of Ophthalmology, Duke University Medical Center, Durham, North Carolina, United States of America; 2 Department of Biomedical Engineering, Duke University Medical Center, Durham, North Carolina, United States of America; 3 Joseph and Kathleen Bryan Alzheimer's Disease Research Center, Durham, North Carolina, United States of America; 4 Department of Medicine, Duke University Medical Center, Durham, North Carolina, United States of America; Charite Universitatsmedizin Berlin, GERMANY

## Abstract

Inner retina in Alzheimer's Disease (AD) may experience neuroinflammation resulting in atrophy. The objective of our study was to determine whether retinal GCIPL (ganglion cell-inner plexiform layer) or nerve fiber layer (NFL) thickness may serve as noninvasive biomarkers to diagnose AD. This cross-sectional case-control study enrolled 15 mild cognitive impairment (MCI) patients, 15 mild-moderate AD patients, and 18 cognitively normal adults. NFL and GCIPL thicknesses on optical coherence tomography (OCT) were measured using Duke Optical Coherence Tomography Retinal Analysis Program (DOCTRAP) and Spectralis software. We demonstrated that regional thicknesses of NFL or GCIPL on macular or nerve OCTs did not differ between groups. However, a multi-variate regression analysis identified macular areas with a significant thickening or thinning in NFL and GCIPL in MCI and AD patients. Our primary findings controvert previous reports of thinner NFL in moderate-to-severe AD. The areas of thickening of GCIPL and NFL in the macula adjacent to areas of thinning, as revealed by a more complex statistical model, suggest that NFL and GCIPL may undergo dynamic changes during AD progression.

## Introduction

Alzheimer’s disease (AD) is the only one of America’s top 10 leading causes of death that has no proven preventive or curative interventions. Early and cost effective diagnosis is crucial to the next stage of treatment and drug development. Current diagnostic modalities for AD are limited by cost (magnetic resonance imaging [MRI] or positron emission tomography [PET]), invasiveness (cerebrospinal fluid [CSF] biomarkers), or suboptimal specificity and sensitivity (genetic markers, serum amyloid) [1]. Neuropsychological evaluation is the “gold standard” for pre-mortem diagnosis of AD [2], but the testing is time-intensive, and may require multiple evaluations or access to specialists. By contrast, optical imaging is an inexpensive, fast, and noninvasive way to view the retina of live patients that may detect neural biomarkers during early stages of AD. Compared to brain imaging modalities, the higher resolution photography achievable with retinal imaging could greatly facilitate early dementia detection [3].

Optical Coherence Tomography (OCT) imaging reveals individual neuronal layers of the retina, including ganglion cell complex layer (GCIPL) and the retinal nerve fiber layer (NFL). Deviation from the age-matched normal range of the thickness for these layers directly correlates with the ganglion cells’ health and is a known biomarker in neurodegenerative diseases such as glaucoma [4], multiple sclerosis [5, 6], or amyotrophic lateral sclerosis [7].

It has long been recognized that patients with early AD experience abnormalities in visual acuity [8, 9], contrast sensitivity [10], color perception [11], visual field [12, 13], and motion perception [14, 15]. Many of the retinal findings associated with AD have been detected early in the disease course and mirror neurodegenerative changes in the brain [16–22]. Javaid and collaborators recently reviewed a dozen of potential visual or ocular markers of Alzheimer’s disease [23]. One of the most promising biomarkers is NFL thickness on OCT. A number of clinical studies have demonstrated quadrant-specific retinal NFL abnormalities in patients with mild cognitive impairment (MCI) or prodromal AD [16–22, 24, 25]. However, the region of the NFL affected varies substantially between these studies. Some have found thinning of NFL in all quadrants surrounding the nerve except nasal[26], others in all quadrants except superior [25], and others only in the temporal [27] or superior quadrant [28]. In contrast, three other studies did not find significant differences in NFL thickness between MCI and cognitively normal controls after carefully excluding potential confounders [29, 30], and in fact a recent study uncovered an inverse relationship between OCT thickness and cognitive scores [30].

The goal of the current study was to resolve this controversy through (1) a carefully designed cross-sectional, case-control study of age-matched, cognitively characterized normal controls, MCI and early-moderate AD patients; and (2) application of two separate tools for OCT layer segmentation. Semi-automatic segmentation methods were employed to precisely quantify NFL and GCIPL thicknesses on OCTs of both the macula and the optic nerve. To our knowledge, our study is the first to have three age-matched cognitive groups with neurocognitive group assignment made by clinical evaluation and consensus diagnosis, as well as careful exclusion of eyes with neovascular age-related macular degeneration (AMD), glaucoma and image artifacts (epiretinal membranes with traction etc.).

## Materials and methods

### Study subjects

The cross-sectional case-control clinical study NCT01937221 at a single academic center enrolled 15 patients with MCI, 15 patients with mild-to-moderate AD, and 18 control subjects who were cognitively normal and age-matched to the patient groups. Written informed consent was obtained from all study participants. 53 subjects were approached from November 14, 2013 and July 8^th^, 2015 and 5 patients screen failed due to the presence of ocular exclusion criteria detailed below. Patients were recruited from the ADRC’s Memory Disorders clinic and included in the mild-to-moderate AD group if they had a diagnosis of probable AD in accordance with the National Institute for Neurological and Communicative Disorders and Stroke–Alzheimer’s Disease and Related Disorders Association (NINCDS-ADRDA) criteria[31] and were deemed to have symptoms characteristic of the mild to moderate stage of the disease. All clinical, imaging, and laboratory data were reviewed by one neurologist (JRB) and one clinical neuropsychologist (GGP) from the Duke Alzheimer’s Disease Research Center (ADRC) to arrive at a consensus decision regarding assignment to cognitive diagnostic group. Patients assigned to the MCI group were deemed to be symptomatic and have predementia, in accordance with published core clinical criteria for MCI [32]. In addition to available diagnoses of cognitive impairment, the clinicians evaluated clinical history for presence of cognitive and functional impairment, and for evidence of change in cognitive or functional impairment over time. Record review included the results of MRI, PET, and laboratory studies collected in the last year, as available. The clinicians viewed these as supportive information rather than as biomarkers. Normal age-matched controls were recruited from the ADRC registries, which monitor cognitive trajectories of individuals in the community. Individuals with normal performance on ADRC registry batteries (MoCA[33], Trail Making B[34], and Delayed Recall from the CERAD Word-listing learning test[35]) were contacted and neurocognitive assessment (Montreal cognitive assessment, MoCA) was repeated at the time of enrollment. Control subjects did not have a history of alcohol abuse, and were not affected by metabolic diseases, psychiatric or neurological disorders that can lead to cognitive decline.

Study inclusion criteria were the following: 1) Clinical review by ADRC clinicians who assigned patients to one of the participant groups, 2) Age ≥ 50 years and matched (+/- 5 years in all but two cases) to a participant in each of the other diagnostic groups, and 3) Fluency in English. Exclusion Criteria were: 1) Known or suspected diagnosis of non-AD, associated dementia, 2) Alcohol or drug addiction in past year or current systemic illness that could influence the patient’s safety and compliance with the protocol, and 3) Major ophthalmologic comorbidities: Ruptured globe, retinal vascular occlusive disease, retinal artery occlusion, anterior ischemic optic neuropathy, media opacification due to corneal abnormalities or cataract that prevent ocular and OCT examination, glaucoma, AMD, and macular edema.

Informed consent was obtained from all the patients or from their legal representatives when appropriate. The research followed the tenets of the Declaration of Helsinki and the protocol was approved by the Institutional Review Board at Duke University Medical Center.

### Ophthalmologic examination

Study participants underwent a complete ophthalmologic examination including assessment of best-corrected visual acuity (BCVA), refraction, ocular motility, pupillary reflexes, intraocular pressure, and a dilated slit lamp ophthalmic exam and binocular indirect ophthalmoscopy. All participants had a corrected visual acuity of 20/40 Snellen or better and IOPs less than 21 mm Hg. The exam was performed by ophthalmologists masked to cognitive status and diagnosis (EML and SWC). Following pupil dilation with 1% tropicamide and 2.5% phenylephrine, ultra-high-resolution SD-OCT (Spectralis OCT, Heidelberg Engineering, Heidelberg, Germany) of both macula and the optic nerve (OCT NFL) and stereo photos of the optic nerve were obtained. All eyes that satisfied the inclusion criteria were included in the analysis.

### NFL/GCIPL thickness measurement

The captured OCT images were enhanced using Spectralis Automatic Real-time Tracking (ART), which resulted in averaging between 7 to 41 B-scans. The macula scan protocol was set to capture 49 line scans for the 20 by 20 degrees scans and 61 line scans for the 30 by 25 degrees scans. For each macular and optic nerve volumetric scan, location-specific NFL and GCIPL thicknesses were measured semi-automatically as previously described using the Duke Optical Coherence Tomography Retinal Analysis Program (DOCTRAP) software that has been validated in numerous large scale clinical trials [36–41]. The software defined 6 retinal sublayers including NFL and GCIPL. GCIPL was defined as the sum of the ganglion cell layer and inner plexiform layer. Automated grading performed by the DOCTRAP software was followed by a two-grader quality control procedure to further examine segmentation boundaries and perform any manual corrections in masked fashion, as previously described [42]. Sub-layer thicknesses were automatically measured in each scan and average macular sub-layer thicknesses were calculated for each volumetric scan.

NFL and GCIPL layers were segmented and thicknesses were measured in all clock hours surrounding the optic nerve (peripapillary) and in all macular regions as defined by the Early Treatment Diabetic Retinopathy Study (ETDRS) [43] (**S1 Fig**). The OCT grader was masked to participant group assignment. The NFL thicknesses in areas surrounding the optic nerve were also generated and analyzed using the Heidelberg automated software. Masked graders checked for segmentation errors made by the Heidelberg automated software and corrected for any errors identified.

The nine-point advised protocol for OCT study terminology and elements (APOSTEL)[44] is presented in **S1 Table.**

### Descriptive statistics

Descriptive statistics were computed for the three study groups for all variables. Demographics were compared using analysis of variance for continuous variables and Fisher’s exact test for categorical variables. Snellen visual acuities were converted to LogMAR units for analysis. Descriptive statistics for measures of thickness were computed by region using both eyes for a given layer (NFL and GCIPL) and OCT image type (macula and nerve). *p* value <0.05 defined statistical significance. Means were compared among and between groups using generalized estimating equations (GEE) to account for multiple eyes per subject. Statistical analyses were performed in SAS 9.3 (SAS Institute Inc., Cary, NC, USA). A sample size of 15 per group (3 groups, with uniform dispersion) provides sufficient power to detect large signals (Beta = 0.83 for effect size = 0.5), as would be ideal to develop a diagnostic test with high clinical value.

### Multivariate regression analysis of NFL/GCIPL thickness

**Fig 1:**
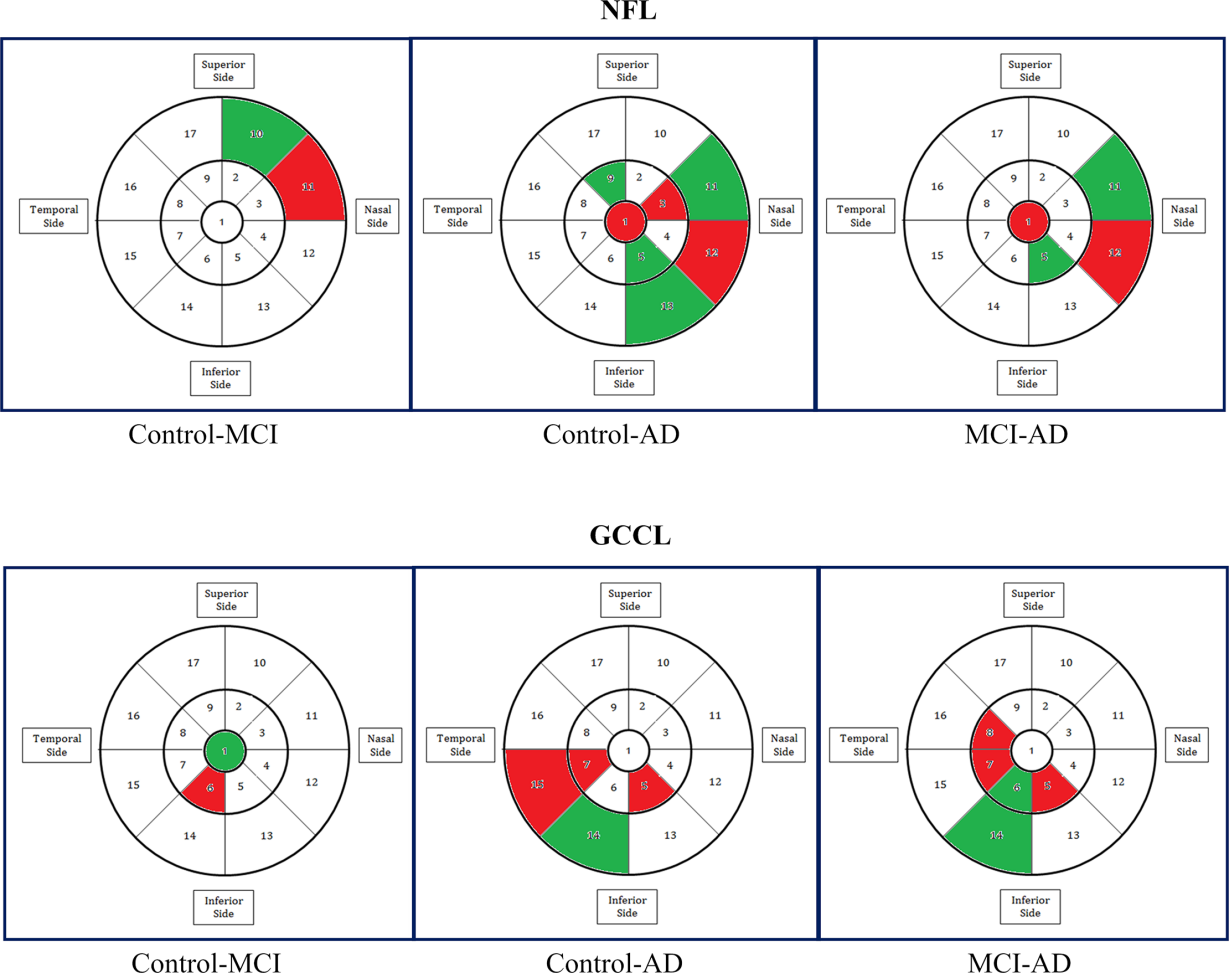
Results of a multi-variate regression analysis with quasi-least squares, without correction for multiple comparisons. This analysis identified areas in the macula that were statistically significantly thinner (red) or thicker (green) in NFL and GCIPL in MCI and AD patients as compared to controls, or in AD compared to MCI.

**Fig 2:**
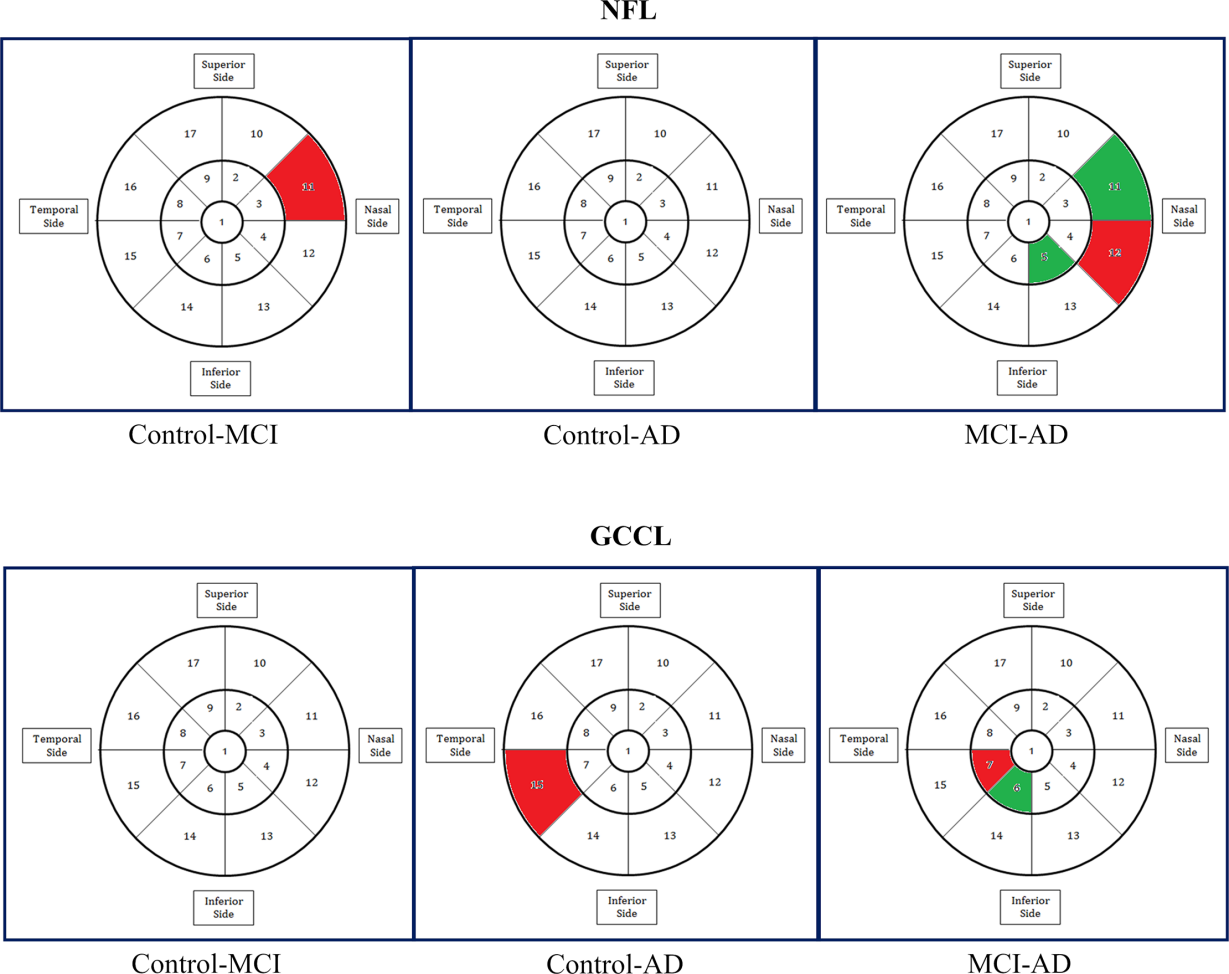
Results of the multi-variate regression analysis with quasi-least squares, adjusted for multiple comparisons.

We used multivariate regression analysis to study the association between the input data (NFL and GCIPL layer thicknesses) to the output data (disease categories of the participants: control, MCI or AD). For this analysis, there were 17 ETDRS regional thickness values for both NFL and GCIPL. We accounted for the presence of two eyes for each participant by using Quasi-Least Squares (QLS) technique [45]. The sign of the coefficient of regression for the input variable represents the direction of association. The associations and directions are pictorially depicted in **Fig 1,** in which associated regions (p<0.05) are colored in green or red according to its direction: increase (green) or decrease (red) in thickness. A Bonferroni correction was then applied to account for multiple comparisons across the 17 areas (p<0.0029), and the regions that remained statistically different were shown in **Fig 2**.

## Results

**Fig 3:**
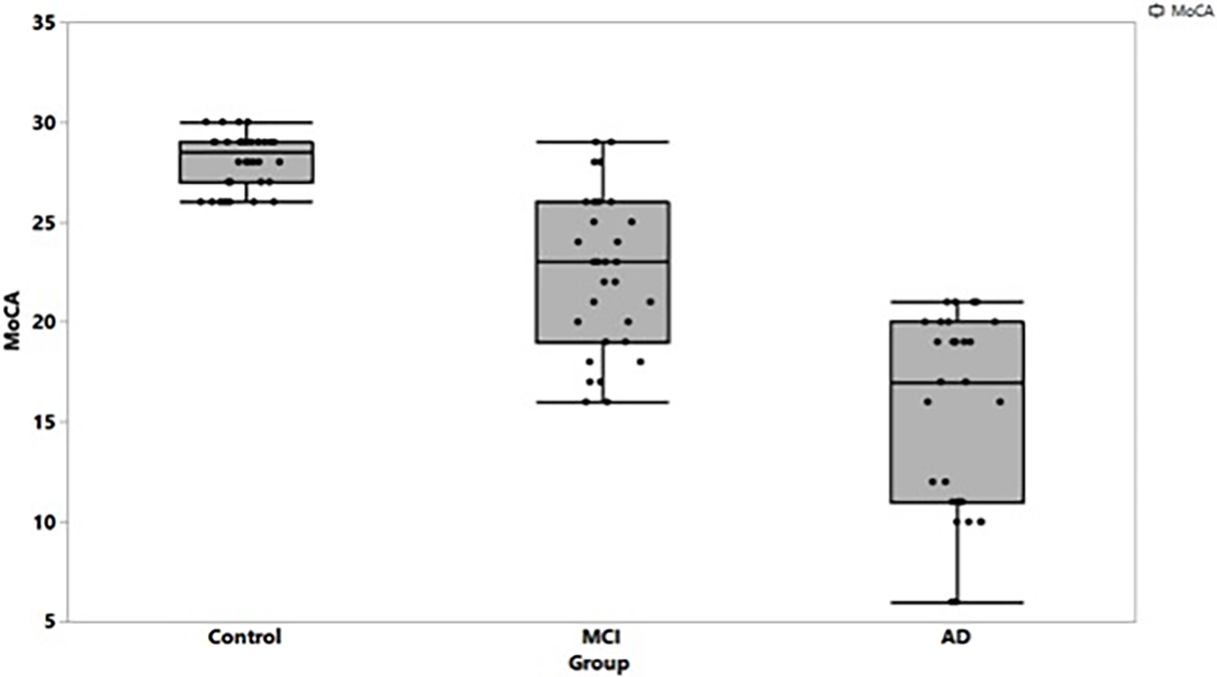
Distribution of MoCA scores among the three study groups.

The three study groups (cognitively normal control group, MCI and mild to-moderate AD groups) were well balanced in terms of age (*p* = 0.79), gender, race (**Table 1**), visual acuity, and intraocular pressure (**S2 Table**). The MCI and AD groups exhibited statistically significantly lower neurocognitive scores than the control subjects (*p*<0.001) (**Table 1**). The distribution of the MoCA scores is depicted in **Fig 3.** Overlap was noted in the range of the MoCA scores between the AD and MCI group and between the MCI and control group, but not between the AD and the control group.

**Table 1:** Demographic characteristics of the cohort.

Variable	Statistic	Alzheimer	Control	MCI	Overall P-Value	Alzheimer vs Control P-Value*	MCI vs Control P-Value*	Alzheimer vs Control P-Value*
**Age**	N	15	18	15				
	Mean (SD, range)	74.20 (8.98, 55–86)	75.17 (5.92, 66–83)	73.07 (9.06, 58–86)				
	Min, Median, Max	55.0, 77.0, 86.0	66.0, 74.5, 83.0	58.0, 75.0, 86.0	0.789*	-	-	-
Male Gender	N (%)	8 (53)	10 (56)	7 (47)	0.872**	-	-	-
Ethnicity, % Caucasian	N (%)	14 (93.3)	14 (93.3)	15 (100)	0.521**	-	-	-
MoCA	N	15	18	15				
	Mean (SD)	15.47 (4.96)	28.06 (1.39)	22.47 (3.96)				
	Min, Median, Max	6.0, 17.0, 21.0	26.0, 28.5, 30.0	16.0, 23.0, 29.0	<0.001*	**<0.001**	**<0.001**	**0.001**

*P-value based on Kruskal-Wallis test of difference among medians or Wilcoxon rank sum test of difference between medians.

**P-value based on chi-square test of difference among proportions.

Comparisons between groups not reported, as omnibus test failed to detect a significant difference across groups (p>0.5).

First, NFL and GCIPL thicknesses measured using semi-automatic segmentation employing DOCTRAP software were compared separately for the various regions on the volumetric OCT macular scans as defined by the ETDRS study [43] and for all of the 12 clock hours surrounding the optic nerve on the volumetric OCT nerve scans. No statistically significant differences were observed between groups in thickness of NFL or GCIPL on OCTs of the macula or the optic nerve. The lack of difference between groups persisted when we compared measures that represented thicknesses of macula or optic nerve globally (**Table 2**), thicknesses of specific ETDRS areas in the macula or clock hours surrounding the optic nerve, or combination measures representing the thickness of larger geographic regions of the macula (inner and outer, superior, inferior, nasal and temporal) after adjusting for multiple comparisons (**S3–S6 Tables**).

**Table 2:** Global GCIPL and NFL thicknesses obtained using the DOCTRAP software in OCT volume scans of the macula and nerve.

Region	Statistic	Alzheimer	Control	MCI	Overall P-Value*	Alzheimer vs Control P-Value*	MCI vs Control P-Value*	Alzheimer vs MCI P-Value*
Global Macula GCCL	N	29	33	23				
	Mean (SD)	64.14 (8.19)	64.08 (3.60)	63.71 (7.12)	0.993	0.979	0.919	0.921
	Min, Median, Max	42.1, 65.5, 76.9	56.3, 65.2, 68.7	45.8, 65.2, 75.3				
Global Macula NFL	N	29	33	23				
	Mean (SD)	39.29 (4.49)	37.71 (3.99)	36.68 (4.22)	0.220	0.328	0.364	0.080
	Min, Median, Max	30.8, 39.4, 50.8	31.3, 36.8, 50.1	27.6, 37.8, 43.3				
Global Nerve GCCL	N	30	36	30				
	Mean (SD)	42.17 (7.68)	40.20 (3.31)	41.28 (4.22)	0.476	0.326	0.359	0.671
	Min, Median, Max	33.6, 41.0, 68.7	33.5, 40.2, 46.3	33.6, 40.9, 52.5				
Global Nerve NFL	N	30	36	30				
	Mean (SD)	100.7 (16.13)	97.15 (8.94)	95.69 (10.92)	0.561	0.409	0.655	0.283
	Min, Median, Max	45.0, 101.9, 129.1	82.7, 96.8, 115.7	69.6, 93.1, 123.0				

*P-values based on test of difference among and between groups using generalized estimating equations (GEE) to account for multiple eyes per subject.

Similarly, no statistically significant differences were observed between groups in thickness of NFL layer surrounding the optic nerve obtained via automated segmentation with the Heidelberg software (**Table 3**).

**Table 3:** NFL thicknesses obtained using the automated Heidelberg software in various regions surrounding the optic nerve.

Variable	Statistic	Alzheimer	Control	MCI	P-Value
Nasal Superior	N	15	18	15	
	Mean (SD)	92.53 (23.51)	85.44 (15.05)	91.33 (19.79)	
	Min, Median, Max	26.0, 90.0, 126.0	60.0, 86.5, 115.0	67.0, 97.0, 136.0	0.347
Nasal	N	15	18	15	
	Mean (SD)	78.27 (22.86)	66.56 (14.57)	68.00 (13.02)	
	Min, Median, Max	46.0, 75.0, 133.0	35.0, 68.0, 91.0	37.0, 68.0, 95.0	0.272
Nasal Inferior	N	15	18	15	
	Mean (SD)	113.7 (33.67)	104.8 (26.95)	98.53 (25.10)	
	Min, Median, Max	70.0, 107.0, 187.0	52.0, 105.5, 156.0	54.0, 97.0, 151.0	0.423
Temporal Superior	N	15	18	15	
	Mean (SD)	129.8 (24.17)	123.9 (16.51)	128.9 (16.23)	
	Min, Median, Max	78.0, 134.0, 167.0	100.0, 122.5, 171.0	94.0, 131.0, 150.0	0.220
Temporal	N	15	18	15	
	Mean (SD)	72.33 (14.72)	70.50 (14.01)	68.27 (14.11)	
	Min, Median, Max	51.0, 76.0, 93.0	49.0, 69.0, 100.0	49.0, 68.0, 97.0	0.634
Temporal Inferior	N	15	18	15	
	Mean (SD)	137.3 (23.20)	131.4 (20.95)	135.5 (15.00)	
	Min, Median, Max	102.0, 144.0, 177.0	97.0, 128.5, 168.0	108.0, 138.0, 157.0	0.648

*P-value based on Kruskal-Wallis test of difference among medians.

The results of an analysis using a multi-variate regression model with quasi-least squares are shown in **Figs 1 and 2**. The results without correction (**Fig 1**) should be interpreted with caution. However, even after correcting for multiple comparisons across the 17 areas analyzed, we identified areas in the macula that were statistically significantly thinner (red) or thicker (green) in NFL and GCIPL layers in MCI and AD patients as compared to cognitively normal controls. It was observed that, when comparing data from MCI or AD to data from controls, areas that were found to be significantly thinner in AD or MCI were often abutting areas found to be significantly thicker in AD or MCI.

## Discussion

Our case-controlled clinical study addressed a major controversy in the field of ocular biomarkers for early diagnosis of AD. We reported two parallel analyses employing different segmentation methods and standard statistical analyses, which both found no statistically significant difference in the thickness of inner retinal layers, NFL and GCIPL, in the macular and optic nerve volumes between cognitively healthy participants and MCI and early-moderate AD patients. This lack of difference was observed after careful exclusion of glaucoma, neovascular AMD and other significant retinal diseases, as well as areas with image artifacts or distortion secondary to retinal processes such as tractional epiretinal membranes. The first segmentation method employed was the DOCTRAP software, which has been used in many prior and ongoing clinical trials to accurately quantify the NFL and GCIPL thicknesses on OCT volumes of macula and optic nerve [42]. The second segmentation software used was that available on the Heidelberg SD-OCT units that quantifies NFL thicknesses in various areas surrounding the optic nerve, as previously done in a number of studies [16–25]. Our negative findings controverted previous reports of thinner NFL in moderate to severe AD patients compared to controls [16–25, 46].

We believe that our different result may be attributed to a number of factors: dynamic changes in the inner retinal layers with progression of MCI and AD[30], variable quality of case identification used in enrolling MCI and AD patients in other studies, or rigor of adjustment for confounders such as glaucoma and retinal artifacts (i.e. epiretinal membranes) that may influence retinal thickness. The current clinical study was robustly designed to address many of the limitations of previous studies, by using a case-control design, creating diagnostic groups balanced in age, race, and gender, and excluding confounding ocular conditions and systemic causes of cognitive decline. Consensus decisions regarding the assignment of the subjects to cognitive diagnostic groups were made by one neurologist one clinical neuropsychologist, both very experienced in diagnosis and management of AD [47]. Because controls were recruited from ADRC registries, they had undergone similar cognitive evaluations as the MCI or AD study participants.

Although our primary finding was the lack of association between NFL and GCIPL thicknesses and MCI or early AD, an interesting and important observation emerged from a more complex statistical analysis performed. Using a multi-variate regression model with quasi-least squares, we demonstrated the existence of specific areas of thickening alternating with areas of thinning in the macula of AD and MCI patients. This finding supports the idea that NFL and GCIPL thickness may be undergoing dynamic changes during the course of AD progression. Indeed, using the Spectralis OCT software, Ferrari and colleagues demonstrated a significant global NFL thinning in moderate AD but not mild AD patients as compared to controls [48], suggesting that thinning of the NFL may not occur until the severe stages of AD. Also in support of this hypothesis, Knoll and colleagues recently reported an inverse relationship between NFL thickness and scores on two neurocognitive tests, a delayed story recall and word list learning test [30]. The retinal thickening in early cognitive impairment was attributed to gliosis (and transient thickening) preceding neuronal loss and atrophy of the axonal projections in the NFL [30]. This concept has been reinforced by histopathology work suggesting that gliosis precedes human AD pathology in the brain [49, 50]. Although current OCT technology cannot differentiate between gliosis and axonal projections in the NFL, new retinal imaging modalities such as adaptive optics scanning laser ophthalmoscope is expected to enable visualization of the various tissue compartments of the retina with high degree of confidence [51]. A longitudinal study is necessary to ascertain whether the dynamic changes in the inner retina are reproducible, and whether specific areas of NFL/GCIPL thickening precede atrophic areas in MCI/AD subjects.

Questions remain about why various stages of AD might be associated with dynamic changes in the retina. Considering that the retina is a developmental outgrowth of the brain, one possibility is that the retina is vulnerable to the same neuroinflammatory injury that causes neurodegenerative disease in the brain. A second hypothesis is that neuronal dysfunction in the brain of a person with AD may lead to nerve loss in the retina via Wallerian-like degeneration. For example, the nucleus basalis of Meynert (NBM) is implicated in early AD and plays an important role in vision, sending projections to primary visual cortex [52–54]. Very early in the course of AD, the NBM undergoes degeneration and decreased acetylcholine production, which could result in decreased activation in visual cortex. In support of this hypothesis, multiple histopathological reports demonstrated retinal ganglion cell loss and optic nerve degeneration in AD patients [55–57] that would have experienced NBM degeneration very early in the disease process.

Our study has several limitations that may affect interpretation of results. One limitation is the small sample size, which limits power to detect small differences between groups. One larger study that reported significantly thinner retinal NFL in AD observed a small standard difference of 0.2 between AD and MCI [46]. If the population difference is similar to that observed difference, a sample size of over 800 is required to achieve 80% power to detect the difference (with 0.05 alpha error). However, we did not observe even a trend in the hypothesized direction using standard statistical analysis, noting that thicker mean NFL was observed in the AD group. Second, we excluded a small number of areas of the retina with prominent epiretinal membranes, which can result in traction and artifactually higher NFL and GCIPL thickness, and the exclusions effectively decreased the number of areas analyzed on the OCT volumetric scans of the macula and nerve. A third limitation may be the careful exclusion of glaucoma in our study and prior others, since open angle and normal tension glaucoma may be closely linked to AD. In a retrospective, propensity-score-matched analysis, Lin and colleagues reported that primary open glaucoma is a significant predictor of AD [58]. Therefore, our exclusion of glaucoma (which was intended to avoid confounding) may have caused us to miss a clinically significant relationship between AD and retinal thinning that is mediated by, rather than confounded by, glaucoma. A case control study of AD patients with and without glaucoma in which NFL and GCIPL OCT layer thicknesses can be compared would be an informative direction of future inquiry. A fourth limitation is that controls were drawn from a community registry, while cases were recruited from a sample of Memory Disorders clinic patients. Nonetheless, the diagnostic approaches were valid for the respective groups, and our confidence in group assignment was bolstered by the cognitive scores obtained at enrollment. Lastly, some of the AD subjects enrolled in our study may have had concomitant cerebral small-vessel disease (SVD), as AD has been reported to present frequently with SVD [59]. SVD is characterized by lacunar infarcts or diffuse white matter lesions on CT and MRI. Our study excluded patients with known or suspected clinical diagnosis of non-AD associated dementia including stroke and vascular dementia. However, although record review of the subjects enrolled included neuroimaging if previously performed, CT and MRI studies were not always available to exclude a diagnosis of SVD. A recent study of over 4000 Korean subjects showed that NFL defects were significantly associated with white matter lesions, although not with lacunar infarctions [60]. Therefore, it is possible that some of the AD subjects enrolled had concomitant SVD, which may have accounted for the macular areas with thinning in NFL on our multi-variate regression analysis. A future larger study should recruit a larger patient group well characterized by neuroimaging that would allow the diagnosis of SVD as well as identification and validation of useful clinical diagnostic endpoints.

Although the focus of this manuscript is analysis of inner retinal layers on SD-OCT, an important future direction is investigation of other potential retinal biomarkers of early AD. It has been long noted that AMD shares several clinical and pathological features with Alzheimer’s disease [61], which include peripheral retinal abnormalities such as drusen and pigment changes seen on color fundus photographs of AD patients [62, 63]. A recent clinical study has shown significant difference in presence of hard drusen in peripheral retina (manually graded on color and autofluorescence fundus images) of 56 AD patients (25.4%) vs. 46 controls (4.2%) [64]. Amyloid-ß plaques were detected in post-mortem retinas of 8 AD patients and five “probable” AD patients, but not in the five age-matched controls [65]. If future work is able to identify a set of retinal biomarkers, or an algorithm based on a combination of biomarkers, that reliably predicts cognitive decline, the impact on clinical care could be immediate. Even before effective therapy is developed, patients with MCI and their families would benefit from reliable prognostic information in terms of their ability to plan for the future (financial decisions, advanced directives, care/residence decisions, etc.). Reliable prognostication and better planning at the individual level could have significant societal benefit, considering the high societal cost of dementia care world-wide [66]. In the longer-term, better diagnostic tools for early AD would likely hasten the development of effective treatments for this devastating disease.

## Supporting information

S1 Fig Examples of segmentation of OCT B-scans of the macula (A) and nerve (B) using DOCTRAP software. NFL is the layer between the red and yellow segmentation lines and GCIPL between the yellow and green lines.
(TIF)

S1 Table Nine-point advised protocol for OCT study terminology and elements checklist. (DOCX)

S2 Table Ophthalmic characteristics of the cohort, all eyes. (DOCX)

S3 Table GCIPL thicknesses obtained using the DOCTRAP software in ETDRS regions of the macula. (DOCX)

S4 Table NFL thicknesses obtained using the DOCTRAP software in ETDRS regions of the macula. (DOCX)

S5 Table GCIPL thicknesses obtained using the DOCTRAP software in clock hours surrounding the optic nerve. (DOCX)

S6 Table NFL thicknesses obtained using the DOCTRAP software in clock hours surrounding the optic nerve. (DOCX)

## Abbreviations

ADRC: Alzheimer’s Disease Research Center
AMD: age-related macular degeneration
BCVA: best corrected visual acuity
CSF: cerebrospinal fluid
DOCTRAP: Duke Optical Coherence Tomography Retinal Analysis Program
ETDRS: Early Treatment Diabetic Retinopathy Study
GCIPL: ganglion cell complex layer
GEE: generalized estimating equations
LogMAR: Logarithm: of the Minimum Angle of Resolution
MCI: mild cognitive impairment
MoCA: Montreal cognitive assessment
MRI: magnetic resonance imaging
NINCDS-ADRDA: National Institute for Neurological and Communicative Disorders and Stroke–Alzheimer’s Disease and Related Disorders Association
NFL: nerve fiber layer
PET: positron emission tomography
QLS: Quasi-Least Squares
SD-OCT: Spectral domain optical coherence tomography
