# Supplementary figures and images for: Evaluation of inner retinal layers as biomarkers in mild cognitive impairment to moderate Alzheimer’s disease
Source: PLoS One. 2018 Feb 8;13(2):e0192646. doi: 10.1371/journal.pone.0192646 (PMC5805310; doi:10.1371/journal.pone.0192646)

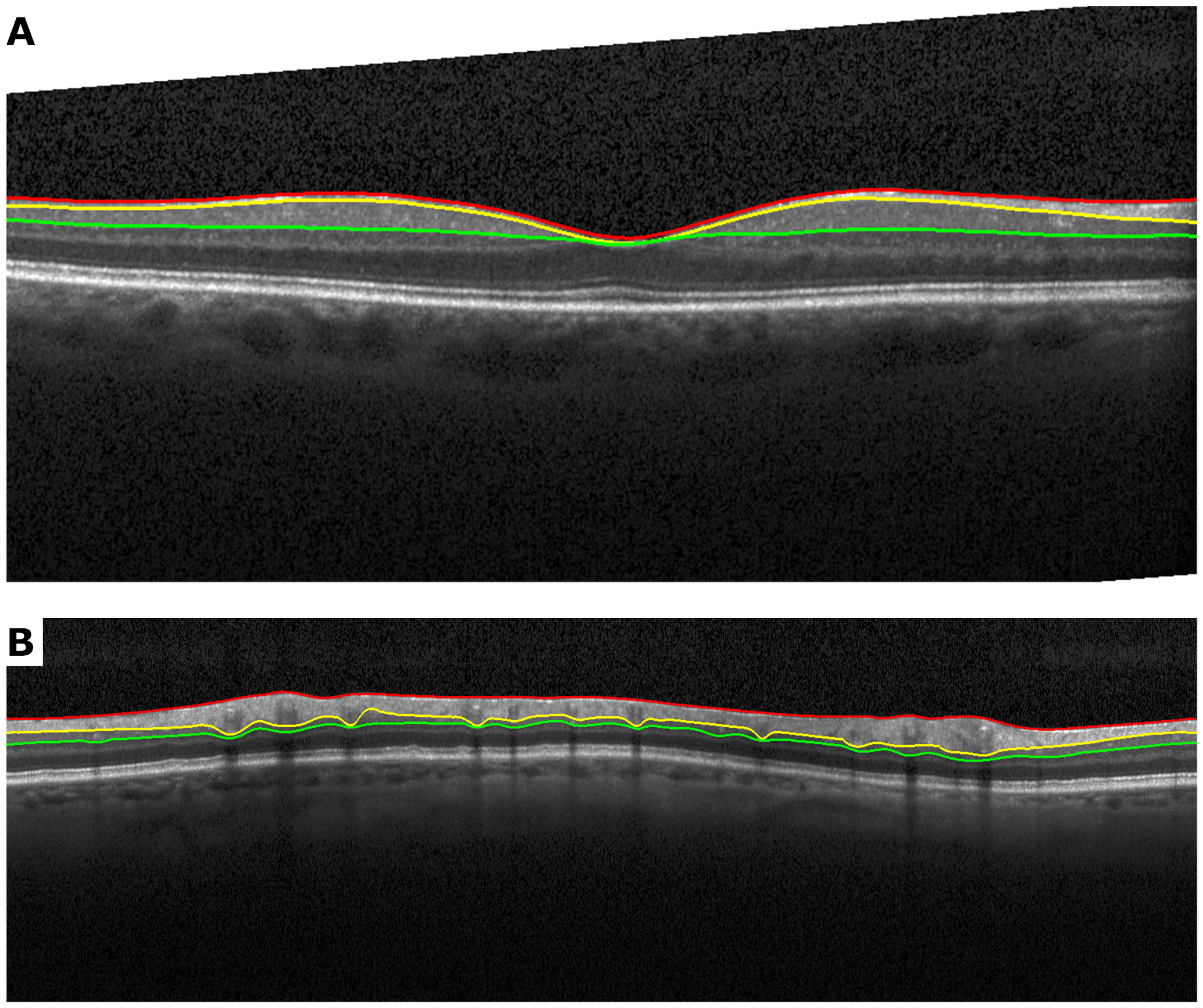

Supplement: S1 Fig — Examples of segmentation of OCT B-scans of the macula (A) and nerve (B) using DOCTRAP software. NFL is the layer between the red and yellow segmentation lines and GCIPL between the yellow and green lines. (TIF) [file pone.0192646.s001.tif]
