# Supplementary material for: Evaluation of inner retinal layers as biomarkers in mild cognitive impairment to moderate Alzheimer’s disease
Source: PLoS One. 2018 Feb 8;13(2):e0192646. doi: 10.1371/journal.pone.0192646 (PMC5805310; doi:10.1371/journal.pone.0192646)
Supplement: S1 Table — (DOCX) [file pone.0192646.s002.docx]

| **Item** | **Recommendation** |
| --- | --- |
| 1 Study Protocol | Describe how many OCT operating sites and graders were included: **one site, single grader** |
|  | Report the timing of OCT compared to other measurements (same day, delayed): **Same day** |
|  | Describe the inclusion and exclusion criteria: **Described on p. 8, lines 171-180.** |
| 2 Acquisition Device | For all OCT devices used, report data on: |
|  | Manufacturer: **Heidelberg Engineering, Heidelberg, Germany** |
|  | Model: **Spectralis HRA+OCT** |
|  | Software version **Heyex 6.3** |
| 3 Acquisition Settings | Clearly describe the settings in which OCT scans were obtained: |
|  | Room light conditions: **dimly lit room** |
|  | Pupils dilated before examination: **yes** |
|  | Number of operators and devices: **one device, two operators** |
| 4 Scanning protocol | Clearly describe the scanning protocol, including: |
|  | Type of scan (circular, volume, star, line, other): **macular volume and circular surrounding optic nerve** |
|  | Location (area of interest, macula, optic nerve head papillomacular bundle, other?): **macula and optic nerve** |
|  | Scan parameters (with or without eye tracking) |
|  | Volume scan: size of scan area (degrees or millimeters), number of B-scans, alignment of B-scans, number of A-scans per B-scan |
|  | Radial scan: size of scan area (degrees of millimeters), number of B-scans, alignment of B-scans, number of A-scans per B-scan |
|  | Ring scan: diameter, A-scan/B-scan, manual or automatic placement of ring or method of centering, depth resolution |
|  | Line scan: angle, location, number of A-scans, depth resolution  **Described on p 9, lines 202-205** |
| 5 Funduscopic imaging | Report other imaging modalities used in addition to OCT (fundoscopy, confocal scanning laser ophthalmoscopy, retinal angiography, autofluorescence imaging): **Stereo photographs of the optic nerve** |
|  | Describe acquisition protocol including**: N/A** |
|  | Excitation wavelength |
|  | Filter sets |
|  | Number of frames averaged (if applicable) |
| 6 Postacquisition data selection | Describe image selection process, including: |
|  | Quality control criteria: **OSCAR-IB criteria** |
|  | Postacquisition discard (number and criteria): **No images were discarded** |
|  | Eye selection strategy (if applicable): **Both eyes were used if they satisfied inclusion criteria** |
| 7 Postacquisition analysis | Describe all postacquisition steps: |
|  | Software used for processing scans and segmentation (may be different from acquisition software): **DOCTRAP software, described on p 9, lines 205-9 and references 35-40** |
|  | Which individual retinal layers were segmented/included: **GCIPL and NFL** |
|  | Method of segmentation (automated, semiautomated, or manually): **semi-automated, described on p 9-10, lined 205-215.** |
|  | How potential bias was addressed in the case of manual segmentation (masking): **The OCT grader was masked to participant group assignment.** |
| 8 Nomenclature and abbreviations | Define: |
|  | Anatomical structures analyzed: **NFL and GCIPL thicknesses** |
|  | Units of provided measurements (e.g., volume or thickness): **thickness in μm** |
| 9 Statistical approach | Describe: |
|  | Statistical models used for the analyses of OCT data: **Described on p 11-12, lines 236-258** |
|  | Whether data were analyzed by eye or by patient: **If they qualified for the study, both eyes were analyzed for each patient** |
