# Supplementary material for: Evaluation of inner retinal layers as biomarkers in mild cognitive impairment to moderate Alzheimer’s disease
Source: PLoS One. 2018 Feb 8;13(2):e0192646. doi: 10.1371/journal.pone.0192646 (PMC5805310; doi:10.1371/journal.pone.0192646)
Supplement: S2 Table — (DOCX) [file pone.0192646.s003.docx]

| **Variable** | **Statistic** | **Alzheimer** | **Control** | **MCI** | **Overall**  **P-Value*** | **Alzheimer**  **vs Control**  **P-Value*** | **MCI**  **vs Control**  **P-Value*** | **Alzheimer**  **vs MCI**  **P-Value*** |
| --- | --- | --- | --- | --- | --- | --- | --- | --- |
| **Visual Acuity (LogMAR)** | N | 30 | 36 | 29 |  |  |  |  |
|  | Mean (SD) | 0.089 (0.107) | 0.049 (0.093) | 0.097 (0.174) | 0.255 | 0.177 | 0.177 | 0.815 |
|  | Min, Median, Max | 0.000, 0.097, 0.398 | -.097, 0.000, 0.301 | 0.000, 0.000, 0.699 |  |  |  |  |
| **IOP** | N | 28 | 36 | 28 |  |  |  |  |
|  | Mean (SD) | 13.8 (2.7) | 15.3 (2.1) | 14.7 (2.9) | 0.242 | 0.093 | 0.503 | 0.360 |
|  | Min, Median, Max | 8, 14, 18 | 12, 16, 20 | 11, 15, 20 |  |  |  |  |

*P-values based on test of difference among and between groups using generalized estimating equations (GEE) to account for multiple eyes per subject.
