# Supplementary material for: Evaluation of inner retinal layers as biomarkers in mild cognitive impairment to moderate Alzheimer’s disease
Source: PLoS One. 2018 Feb 8;13(2):e0192646. doi: 10.1371/journal.pone.0192646 (PMC5805310; doi:10.1371/journal.pone.0192646)
Supplement: S3 Table — (DOCX) [file pone.0192646.s004.docx]

| **Variable** | **Statistic** | **Alzheimer** | **Control** | **MCI** | **Overall**  **P-Value*** | **Alzheimer**  **vs Control**  **P-Value*** | **MCI**  **vs Control**  **P-Value*** | **Alzheimer**  **vs MCI**  **P-Value*** |
| --- | --- | --- | --- | --- | --- | --- | --- | --- |
| Region 1 | N | 29 | 33 | 23 |  |  |  |  |
|  | Mean (SD) | 37.17 (8.70) | 36.16 (12.61) | 37.43 (9.63) | 0.946 | 0.790 | 0.748 | 0.920 |
|  | Min, Median, Max | 21.4, 36.3, 67.7 | 16.0, 36.6, 68.0 | 24.5, 34.3, 63.3 |  |  |  |  |
| Region 2 | N | 29 | 33 | 23 |  |  |  |  |
|  | Mean (SD) | 85.30 (13.19) | 88.13 (6.12) | 85.12 (12.77) | 0.521 | 0.390 | 0.362 | 0.922 |
|  | Min, Median, Max | 35.3, 88.4, 101.3 | 74.2, 89.8, 99.4 | 50.1, 88.1, 102.3 |  |  |  |  |
| Region 3 | N | 29 | 33 | 23 |  |  |  |  |
|  | Mean (SD) | 82.98 (17.88) | 86.97 (7.77) | 85.02 (13.37) | 0.542 | 0.289 | 0.589 | 0.663 |
|  | Min, Median, Max | 2.2, 87.9, 99.2 | 70.6, 87.8, 97.7 | 46.0, 87.4, 102.7 |  |  |  |  |
| Region 4 | N | 29 | 33 | 23 |  |  |  |  |
|  | Mean (SD) | 83.67 (17.28) | 87.07 (7.37) | 85.34 (14.15) | 0.628 | 0.352 | 0.631 | 0.738 |
|  | Min, Median, Max | 8.8, 88.0, 99.6 | 71.3, 86.3, 99.6 | 42.1, 88.5, 106.4 |  |  |  |  |
| Region 5 | N | 29 | 33 | 23 |  |  |  |  |
|  | Mean (SD) | 84.24 (10.96) | 87.27 (6.76) | 84.03 (12.15) | 0.466 | 0.322 | 0.324 | 0.923 |
|  | Min, Median, Max | 52.7, 86.2, 98.4 | 69.3, 88.1, 96.8 | 50.5, 87.1, 102.6 |  |  |  |  |
| Region 6 | N | 29 | 33 | 23 |  |  |  |  |
|  | Mean (SD) | 86.60 (10.22) | 88.71 (5.50) | 85.91 (12.32) | 0.496 | 0.384 | 0.345 | 0.896 |
|  | Min, Median, Max | 66.3, 89.4, 99.6 | 72.5, 89.1, 96.5 | 53.3, 88.2, 106.6 |  |  |  |  |
| Region 7 | N | 29 | 33 | 23 |  |  |  |  |
|  | Mean (SD) | 83.65 (9.92) | 85.10 (5.74) | 84.45 (12.84) | 0.794 | 0.512 | 0.821 | 0.775 |
|  | Min, Median, Max | 56.9, 84.7, 99.2 | 70.1, 84.3, 94.8 | 49.1, 87.7, 106.5 |  |  |  |  |
| Region 8 | N | 29 | 33 | 23 |  |  |  |  |
|  | Mean (SD) | 81.25 (9.08) | 81.06 (7.14) | 81.35 (11.30) | 0.976 | 0.995 | 0.844 | 0.807 |
|  | Min, Median, Max | 63.4, 83.1, 97.9 | 58.6, 81.5, 92.4 | 50.4, 82.1, 100.4 |  |  |  |  |
| Region 9 | N | 29 | 33 | 23 |  |  |  |  |
|  | Mean (SD) | 85.60 (9.77) | 86.53 (6.21) | 84.14 (12.10) | 0.747 | 0.708 | 0.466 | 0.733 |
|  | Min, Median, Max | 67.2, 87.7, 104.0 | 68.7, 88.0, 96.8 | 51.2, 86.7, 101.3 |  |  |  |  |
| Region 10 | N | 29 | 33 | 23 |  |  |  |  |
|  | Mean (SD) | 56.18 (10.13) | 56.41 (4.13) | 57.18 (5.84) | 0.839 | 0.934 | 0.580 | 0.683 |
|  | Min, Median, Max | 20.0, 55.8, 74.7 | 44.1, 57.1, 62.7 | 43.6, 56.4, 71.6 |  |  |  |  |
| Region 11 | N | 29 | 33 | 23 |  |  |  |  |
|  | Mean (SD) | 60.28 (14.46) | 60.91 (5.86) | 60.44 (8.56) | 0.969 | 0.842 | 0.854 | 0.955 |
|  | Min, Median, Max | 0.7, 61.2, 79.2 | 44.8, 61.4, 76.2 | 45.3, 61.7, 75.5 |  |  |  |  |
| Region 12 | N | 29 | 33 | 23 |  |  |  |  |
|  | Mean (SD) | 59.95 (14.31) | 59.56 (4.93) | 59.17 (7.42) | 0.974 | 0.884 | 0.896 | 0.822 |
|  | Min, Median, Max | 0.6, 60.8, 77.4 | 48.9, 59.4, 73.9 | 44.1, 60.9, 75.0 |  |  |  |  |
| Region 13 | N | 29 | 33 | 23 |  |  |  |  |
|  | Mean (SD) | 54.46 (8.08) | 53.82 (4.27) | 53.52 (5.12) | 0.940 | 0.733 | 0.886 | 0.764 |
|  | Min, Median, Max | 27.3, 53.6, 66.5 | 46.1, 54.9, 61.2 | 44.8, 53.1, 63.1 |  |  |  |  |
| Region 14 | N | 29 | 33 | 23 |  |  |  |  |
|  | Mean (SD) | 57.13 (7.06) | 56.25 (4.21) | 55.93 (5.67) | 0.864 | 0.594 | 0.914 | 0.676 |
|  | Min, Median, Max | 43.3, 55.4, 70.1 | 49.5, 57.7, 63.4 | 45.5, 55.6, 65.6 |  |  |  |  |
| Region 15 | N | 29 | 33 | 23 |  |  |  |  |
|  | Mean (SD) | 63.86 (9.31) | 63.25 (4.10) | 62.61 (7.07) | 0.940 | 0.830 | 0.793 | 0.737 |
|  | Min, Median, Max | 40.5, 65.2, 79.0 | 54.2, 62.1, 71.0 | 47.2, 61.5, 72.8 |  |  |  |  |
| Region 16 | N | 29 | 33 | 23 |  |  |  |  |
|  | Mean (SD) | 62.74 (7.45) | 60.99 (3.67) | 61.12 (7.04) | 0.699 | 0.401 | 0.790 | 0.653 |
|  | Min, Median, Max | 48.2, 64.2, 75.8 | 53.1, 60.7, 70.3 | 49.3, 59.5, 72.6 |  |  |  |  |
| Region 17 | N | 29 | 33 | 23 |  |  |  |  |
|  | Mean (SD) | 58.16 (7.60) | 56.64 (3.70) | 57.46 (6.56) | 0.751 | 0.508 | 0.628 | 0.807 |
|  | Min, Median, Max | 47.2, 58.3, 75.8 | 49.5, 56.9, 63.2 | 45.3, 57.1, 72.5 |  |  |  |  |
| Superior | N | 29 | 33 | 23 |  |  |  |  |
|  | Mean (SD) | 71.31 (9.02) | 71.93 (4.49) | 70.97 (8.75) | 0.906 | 0.801 | 0.684 | 0.913 |
|  | Min, Median, Max | 47.8, 72.0, 85.5 | 60.5, 73.3, 79.0 | 47.5, 73.9, 86.4 |  |  |  |  |
| Inferior | N | 29 | 33 | 23 |  |  |  |  |
|  | Mean (SD) | 70.61 (8.46) | 71.51 (4.59) | 69.85 (8.15) | 0.771 | 0.696 | 0.498 | 0.827 |
|  | Min, Median, Max | 50.1, 71.1, 82.4 | 59.6, 72.6, 77.2 | 48.5, 72.7, 83.5 |  |  |  |  |
| Temporal | N | 29 | 33 | 23 |  |  |  |  |
|  | Mean (SD) | 72.88 (8.18) | 72.60 (4.11) | 72.38 (8.86) | 1.000 | 0.978 | 1.000 | 0.999 |
|  | Min, Median, Max | 54.2, 74.6, 84.6 | 63.1, 72.4, 79.7 | 49.1, 74.5, 86.2 |  |  |  |  |
| Nasal | N | 29 | 33 | 23 |  |  |  |  |
|  | Mean (SD) | 71.72 (15.52) | 73.63 (5.06) | 72.49 (10.19) | 0.807 | 0.568 | 0.679 | 0.850 |
|  | Min, Median, Max | 3.1, 73.9, 86.3 | 62.0, 75.2, 82.6 | 44.4, 75.3, 86.8 |  |  |  |  |
| Outer Superior | N | 29 | 33 | 23 |  |  |  |  |
|  | Mean (SD) | 57.17 (8.11) | 56.53 (3.58) | 57.32 (5.98) | 0.839 | 0.779 | 0.580 | 0.912 |
|  | Min, Median, Max | 39.4, 56.2, 74.7 | 47.2, 56.8, 62.0 | 44.4, 56.3, 71.0 |  |  |  |  |
| Inner Superior | N | 29 | 33 | 23 |  |  |  |  |
|  | Mean (SD) | 85.45 (10.75) | 87.33 (6.00) | 84.63 (12.38) | 0.628 | 0.516 | 0.407 | 0.817 |
|  | Min, Median, Max | 56.2, 87.8, 102.7 | 73.7, 88.8, 98.1 | 50.7, 87.4, 101.8 |  |  |  |  |
| Outer Temporal | N | 29 | 33 | 23 |  |  |  |  |
|  | Mean (SD) | 63.30 (8.22) | 62.12 (3.67) | 61.87 (6.91) | 0.880 | 0.614 | 0.997 | 0.694 |
|  | Min, Median, Max | 46.9, 65.4, 77.4 | 54.9, 61.6, 70.7 | 48.5, 60.3, 70.6 |  |  |  |  |
| Inner Temporal | N | 29 | 33 | 23 |  |  |  |  |
|  | Mean (SD) | 82.45 (9.13) | 83.08 (5.88) | 82.90 (11.86) | 0.928 | 0.717 | 0.993 | 0.775 |
|  | Min, Median, Max | 61.6, 84.1, 97.5 | 70.9, 82.9, 92.5 | 49.7, 85.0, 103.4 |  |  |  |  |
| Inner Nasal | N | 29 | 33 | 23 |  |  |  |  |
|  | Mean (SD) | 83.32 (17.49) | 87.02 (7.45) | 85.18 (13.71) | 0.580 | 0.316 | 0.608 | 0.698 |
|  | Min, Median, Max | 5.5, 87.4, 98.4 | 72.2, 86.8, 98.2 | 44.0, 88.7, 104.5 |  |  |  |  |
| Outer Nasal | N | 29 | 33 | 23 |  |  |  |  |
|  | Mean (SD) | 60.11 (14.32) | 60.23 (5.17) | 59.80 (7.92) | 0.986 | 0.977 | 0.871 | 0.935 |
|  | Min, Median, Max | 0.6, 61.7, 77.5 | 49.5, 60.4, 75.0 | 44.7, 61.8, 74.7 |  |  |  |  |
| Inner Inferior | N | 29 | 33 | 23 |  |  |  |  |
|  | Mean (SD) | 85.42 (10.32) | 87.99 (6.03) | 84.97 (12.00) | 0.468 | 0.346 | 0.321 | 0.901 |
|  | Min, Median, Max | 61.2, 86.8, 98.5 | 70.9, 88.5, 96.5 | 51.9, 88.1, 103.6 |  |  |  |  |
| Outer Inferior | N | 29 | 33 | 23 |  |  |  |  |
|  | Mean (SD) | 55.79 (7.20) | 55.03 (4.06) | 54.73 (5.30) | 0.900 | 0.653 | 0.898 | 0.723 |
|  | Min, Median, Max | 39.1, 54.5, 68.3 | 48.3, 56.6, 61.6 | 45.2, 55.5, 63.6 |  |  |  |  |

**P-values based on test of difference among and between groups using generalized estimating equations (GEE) to account for multiple eyes per subject.*
