# Supplementary material for: Evaluation of inner retinal layers as biomarkers in mild cognitive impairment to moderate Alzheimer’s disease
Source: PLoS One. 2018 Feb 8;13(2):e0192646. doi: 10.1371/journal.pone.0192646 (PMC5805310; doi:10.1371/journal.pone.0192646)
Supplement: S4 Table — (DOCX) [file pone.0192646.s005.docx]

| **Variable** | **Statistic** | **Alzheimer** | **Control** | **MCI** | **Overall**  **P-Value*** | **Alzheimer**  **vs Control**  **P-Value*** | **MCI**  **vs Control**  **P-Value*** | **Alzheimer**  **vs MCI**  **P-Value*** |
| --- | --- | --- | --- | --- | --- | --- | --- | --- |
| Region 1 | N | 29 | 33 | 23 |  |  |  |  |
|  | Mean (SD) | 18.65 (8.07) | 17.01 (1.98) | 17.66 (1.30) | 0.321 | 0.292 | 0.227 | 0.511 |
|  | Min, Median, Max | 11.9, 17.4, 59.2 | 11.3, 17.1, 20.4 | 13.6, 18.0, 19.0 |  |  |  |  |
| Region 2 | N | 29 | 33 | 23 |  |  |  |  |
|  | Mean (SD) | 33.41 (8.36) | 30.71 (3.37) | 29.12 (3.30) | 0.100 | 0.218 | 0.124 | **0.047** |
|  | Min, Median, Max | 25.5, 31.3, 70.4 | 26.1, 29.7, 40.3 | 24.8, 28.4, 37.1 |  |  |  |  |
| Region 3 | N | 29 | 33 | 23 |  |  |  |  |
|  | Mean (SD) | 27.52 (3.10) | 27.05 (3.19) | 25.86 (1.49) | 0.063 | 0.609 | 0.086 | **0.043** |
|  | Min, Median, Max | 23.4, 27.0, 35.8 | 21.8, 26.6, 34.8 | 23.8, 25.5, 29.1 |  |  |  |  |
| Region 4 | N | 29 | 33 | 23 |  |  |  |  |
|  | Mean (SD) | 27.85 (4.44) | 26.99 (2.59) | 26.34 (1.67) | 0.251 | 0.447 | 0.266 | 0.142 |
|  | Min, Median, Max | 22.2, 26.6, 44.7 | 22.1, 27.0, 34.0 | 23.8, 26.3, 30.3 |  |  |  |  |
| Region 5 | N | 29 | 33 | 23 |  |  |  |  |
|  | Mean (SD) | 34.31 (9.69) | 31.98 (2.56) | 30.95 (3.87) | 0.345 | 0.279 | 0.367 | 0.156 |
|  | Min, Median, Max | 26.6, 32.7, 80.5 | 28.6, 31.2, 37.1 | 20.8, 31.2, 37.0 |  |  |  |  |
| Region 6 | N | 29 | 33 | 23 |  |  |  |  |
|  | Mean (SD) | 30.79 (7.29) | 28.86 (2.54) | 27.91 (2.90) | 0.313 | 0.281 | 0.337 | 0.142 |
|  | Min, Median, Max | 24.1, 30.0, 64.3 | 25.5, 28.8, 35.4 | 20.1, 27.8, 33.0 |  |  |  |  |
| Region 7 | N | 29 | 33 | 23 |  |  |  |  |
|  | Mean (SD) | 24.09 (3.08) | 23.56 (1.29) | 23.08 (1.26) | 0.284 | 0.399 | 0.312 | 0.135 |
|  | Min, Median, Max | 21.8, 23.3, 38.9 | 21.8, 23.4, 27.9 | 19.8, 22.8, 25.3 |  |  |  |  |
| Region 8 | N | 29 | 33 | 23 |  |  |  |  |
|  | Mean (SD) | 23.54 (2.53) | 22.78 (1.01) | 22.50 (1.46) | 0.260 | 0.190 | 0.434 | 0.101 |
|  | Min, Median, Max | 21.1, 23.1, 35.7 | 20.9, 22.9, 24.9 | 17.3, 22.6, 25.1 |  |  |  |  |
| Region 9 | N | 29 | 33 | 23 |  |  |  |  |
|  | Mean (SD) | 30.49 (5.57) | 28.32 (2.83) | 27.36 (2.80) | 0.129 | 0.201 | 0.233 | **0.050** |
|  | Min, Median, Max | 25.4, 29.7, 52.6 | 24.5, 27.4, 37.5 | 23.1, 26.5, 34.6 |  |  |  |  |
| Region 10 | N | 29 | 33 | 23 |  |  |  |  |
|  | Mean (SD) | 54.05 (10.82) | 49.54 (6.03) | 48.12 (6.83) | 0.191 | 0.160 | 0.473 | 0.069 |
|  | Min, Median, Max | 36.3, 53.4, 93.0 | 38.2, 49.8, 65.9 | 32.5, 49.1, 60.5 |  |  |  |  |
| Region 11 | N | 29 | 33 | 23 |  |  |  |  |
|  | Mean (SD) | 52.41 (12.35) | 53.13 (7.35) | 48.95 (8.10) | 0.235 | 0.780 | 0.092 | 0.231 |
|  | Min, Median, Max | 0.6, 53.6, 67.6 | 42.5, 52.1, 77.1 | 33.5, 49.5, 63.3 |  |  |  |  |
| Region 12 | N | 29 | 33 | 23 |  |  |  |  |
|  | Mean (SD) | 56.20 (8.38) | 57.03 (10.86) | 55.32 (12.64) | 0.793 | 0.726 | 0.487 | 0.647 |
|  | Min, Median, Max | 36.1, 56.6, 69.1 | 41.6, 53.1, 87.4 | 33.0, 52.5, 82.4 |  |  |  |  |
| Region 13 | N | 29 | 33 | 23 |  |  |  |  |
|  | Mean (SD) | 56.26 (12.81) | 51.67 (9.58) | 53.17 (11.66) | 0.445 | 0.218 | 0.963 | 0.319 |
|  | Min, Median, Max | 33.6, 55.3, 102.4 | 38.4, 50.2, 77.3 | 34.6, 52.3, 78.2 |  |  |  |  |
| Region 14 | N | 29 | 33 | 23 |  |  |  |  |
|  | Mean (SD) | 38.76 (5.67) | 36.75 (4.25) | 36.02 (4.66) | 0.239 | 0.246 | 0.489 | 0.090 |
|  | Min, Median, Max | 27.4, 39.2, 53.0 | 30.5, 36.2, 52.0 | 26.0, 35.8, 43.4 |  |  |  |  |
| Region 15 | N | 29 | 33 | 23 |  |  |  |  |
|  | Mean (SD) | 26.42 (3.43) | 25.40 (1.78) | 25.06 (1.60) | 0.308 | 0.239 | 0.517 | 0.126 |
|  | Min, Median, Max | 23.0, 25.5, 38.8 | 22.7, 25.0, 30.6 | 23.1, 25.1, 30.5 |  |  |  |  |
| Region 16 | N | 29 | 33 | 23 |  |  |  |  |
|  | Mean (SD) | 24.59 (1.91) | 24.01 (1.26) | 23.29 (1.75) | 0.228 | 0.302 | 0.187 | 0.091 |
|  | Min, Median, Max | 21.5, 23.8, 29.4 | 21.8, 24.0, 26.4 | 18.3, 23.2, 27.7 |  |  |  |  |
| Region 17 | N | 29 | 33 | 23 |  |  |  |  |
|  | Mean (SD) | 36.91 (5.96) | 34.64 (3.91) | 33.34 (3.96) | 0.223 | 0.230 | 0.351 | 0.089 |
|  | Min, Median, Max | 27.9, 36.2, 54.4 | 26.9, 34.7, 42.1 | 28.5, 32.7, 40.5 |  |  |  |  |
| Superior | N | 29 | 33 | 23 |  |  |  |  |
|  | Mean (SD) | 38.72 (7.17) | 35.80 (3.50) | 34.49 (3.73) | 0.128 | 0.163 | 0.253 | **0.045** |
|  | Min, Median, Max | 29.3, 38.0, 67.6 | 30.0, 35.3, 44.9 | 27.3, 34.4, 42.8 |  |  |  |  |
| Inferior | N | 29 | 33 | 23 |  |  |  |  |
|  | Mean (SD) | 40.03 (8.26) | 37.32 (4.35) | 37.01 (5.23) | 0.345 | 0.208 | 0.693 | 0.153 |
|  | Min, Median, Max | 28.3, 39.8, 75.1 | 30.8, 36.6, 50.3 | 25.3, 37.6, 46.7 |  |  |  |  |
| Temporal | N | 29 | 33 | 23 |  |  |  |  |
|  | Mean (SD) | 24.66 (2.44) | 23.94 (0.97) | 23.48 (1.13) | 0.184 | 0.222 | 0.199 | 0.074 |
|  | Min, Median, Max | 22.7, 23.9, 35.7 | 22.1, 23.7, 26.6 | 21.2, 23.5, 25.8 |  |  |  |  |
| Nasal | N | 29 | 33 | 23 |  |  |  |  |
|  | Mean (SD) | 41.00 (4.90) | 41.05 (5.24) | 39.12 (5.33) | 0.392 | 0.943 | 0.201 | 0.215 |
|  | Min, Median, Max | 28.7, 41.6, 49.6 | 32.8, 39.8, 55.6 | 30.1, 38.0, 48.2 |  |  |  |  |
| Outer Superior | N | 29 | 33 | 23 |  |  |  |  |
|  | Mean (SD) | 45.48 (8.18) | 42.09 (4.74) | 40.73 (5.04) | 0.184 | 0.176 | 0.398 | 0.067 |
|  | Min, Median, Max | 32.1, 45.4, 73.7 | 33.9, 41.9, 54.0 | 30.5, 41.2, 50.4 |  |  |  |  |
| Inner Superior | N | 29 | 33 | 23 |  |  |  |  |
|  | Mean (SD) | 31.95 (6.83) | 29.51 (3.02) | 28.24 (2.97) | 0.104 | 0.202 | 0.156 | **0.044** |
|  | Min, Median, Max | 25.5, 30.3, 61.5 | 26.0, 28.8, 38.9 | 24.2, 27.7, 35.3 |  |  |  |  |
| Outer Temporal | N | 29 | 33 | 23 |  |  |  |  |
|  | Mean (SD) | 25.51 (2.36) | 24.71 (1.29) | 24.17 (1.45) | 0.214 | 0.230 | 0.264 | 0.083 |
|  | Min, Median, Max | 22.9, 24.9, 34.1 | 22.4, 24.4, 28.5 | 21.4, 23.8, 28.0 |  |  |  |  |
| Inner Temporal | N | 29 | 33 | 23 |  |  |  |  |
|  | Mean (SD) | 23.82 (2.74) | 23.17 (0.97) | 22.79 (1.24) | 0.259 | 0.266 | 0.316 | 0.108 |
|  | Min, Median, Max | 21.9, 23.4, 37.3 | 21.8, 23.1, 25.8 | 18.5, 22.7, 24.6 |  |  |  |  |
| Inner Nasal | N | 29 | 33 | 23 |  |  |  |  |
|  | Mean (SD) | 27.69 (3.57) | 27.02 (2.74) | 26.10 (1.44) | 0.115 | 0.504 | 0.126 | 0.075 |
|  | Min, Median, Max | 23.4, 26.8, 39.1 | 22.0, 26.4, 33.9 | 23.8, 25.7, 28.9 |  |  |  |  |
| Outer Nasal | N | 29 | 33 | 23 |  |  |  |  |
|  | Mean (SD) | 54.31 (9.67) | 55.08 (8.81) | 52.14 (9.64) | 0.548 | 0.737 | 0.256 | 0.427 |
|  | Min, Median, Max | 18.3, 55.8, 66.9 | 42.2, 53.7, 80.3 | 33.2, 52.1, 67.7 |  |  |  |  |
| Inner Inferior | N | 29 | 33 | 23 |  |  |  |  |
|  | Mean (SD) | 32.55 (8.46) | 30.42 (2.43) | 29.43 (3.30) | 0.321 | 0.278 | 0.346 | 0.147 |
|  | Min, Median, Max | 25.7, 31.6, 72.4 | 27.1, 30.2, 36.0 | 20.4, 29.4, 34.7 |  |  |  |  |
| Outer Inferior | N | 29 | 33 | 23 |  |  |  |  |
|  | Mean (SD) | 47.51 (8.96) | 44.21 (6.66) | 44.60 (7.76) | 0.381 | 0.215 | 0.863 | 0.208 |
|  | Min, Median, Max | 30.5, 47.4, 77.7 | 34.5, 42.9, 64.7 | 30.3, 44.0, 59.9 |  |  |  |  |

'*P-values based on test of difference among and between groups using generalized estimating equations (GEE) to account for multiple eyes per subject.
