# Supplementary material for: Evaluation of inner retinal layers as biomarkers in mild cognitive impairment to moderate Alzheimer’s disease
Source: PLoS One. 2018 Feb 8;13(2):e0192646. doi: 10.1371/journal.pone.0192646 (PMC5805310; doi:10.1371/journal.pone.0192646)
Supplement: S5 Table — (DOCX) [file pone.0192646.s006.docx]

| **Variable** | **Statistic** | **Alzheimer** | **Control** | **MCI** | **Overall**  **P-Value*** | **Alzheimer**  **vs Control**  **P-Value*** | **MCI**  **vs Control**  **P-Value*** | **Alzheimer**  **vs MCI**  **P-Value*** |
| --- | --- | --- | --- | --- | --- | --- | --- | --- |
| Region 1 | N | 30 | 36 | 30 |  |  |  |  |
|  | Mean (SD) | 41.32 (12.82) | 36.71 (7.41) | 39.73 (5.73) | 0.216 | 0.160 | 0.131 | 0.607 |
|  | Min, Median, Max | 26.4, 39.9, 98.3 | 21.3, 37.1, 53.3 | 31.1, 39.8, 60.4 |  |  |  |  |
| Region 2 | N | 30 | 36 | 30 |  |  |  |  |
|  | Mean (SD) | 39.97 (12.12) | 35.73 (8.90) | 37.83 (5.81) | 0.382 | 0.201 | 0.282 | 0.468 |
|  | Min, Median, Max | 24.7, 37.4, 87.3 | 18.2, 35.2, 53.9 | 25.3, 37.0, 56.6 |  |  |  |  |
| Region 3 | N | 30 | 36 | 30 |  |  |  |  |
|  | Mean (SD) | 41.61 (8.35) | 39.35 (6.81) | 40.95 (8.71) | 0.539 | 0.282 | 0.471 | 0.773 |
|  | Min, Median, Max | 31.9, 39.6, 75.8 | 25.5, 40.1, 58.5 | 25.3, 40.5, 70.0 |  |  |  |  |
| Region 4 | N | 30 | 36 | 30 |  |  |  |  |
|  | Mean (SD) | 41.34 (9.36) | 38.93 (5.72) | 41.23 (6.31) | 0.241 | 0.288 | 0.124 | 0.962 |
|  | Min, Median, Max | 23.4, 40.8, 68.0 | 28.7, 38.2, 53.8 | 25.2, 41.1, 56.1 |  |  |  |  |
| Region 5 | N | 30 | 36 | 30 |  |  |  |  |
|  | Mean (SD) | 37.94 (12.73) | 38.57 (6.46) | 38.68 (6.71) | 0.968 | 0.822 | 0.954 | 0.802 |
|  | Min, Median, Max | 21.4, 35.0, 94.9 | 29.0, 38.5, 53.7 | 22.3, 39.8, 49.5 |  |  |  |  |
| Region 6 | N | 30 | 36 | 30 |  |  |  |  |
|  | Mean (SD) | 39.89 (17.30) | 37.97 (8.91) | 39.87 (9.17) | 0.662 | 0.595 | 0.395 | 0.995 |
|  | Min, Median, Max | 24.1, 37.0, 124.1 | 27.2, 36.3, 81.2 | 29.4, 39.3, 79.8 |  |  |  |  |
| Region 7 | N | 30 | 36 | 30 |  |  |  |  |
|  | Mean (SD) | 38.45 (11.98) | 35.97 (7.78) | 38.09 (6.20) | 0.406 | 0.417 | 0.208 | 0.905 |
|  | Min, Median, Max | 21.6, 35.7, 86.6 | 22.0, 34.8, 59.7 | 27.5, 38.0, 57.1 |  |  |  |  |
| Region 8 | N | 30 | 36 | 30 |  |  |  |  |
|  | Mean (SD) | 43.64 (12.50) | 44.53 (6.75) | 42.20 (9.61) | 0.563 | 0.672 | 0.290 | 0.587 |
|  | Min, Median, Max | 2.3, 42.7, 75.0 | 30.6, 44.8, 56.8 | 29.7, 39.6, 76.9 |  |  |  |  |
| Region 9 | N | 30 | 36 | 30 |  |  |  |  |
|  | Mean (SD) | 51.55 (12.48) | 54.03 (7.13) | 53.79 (9.95) | 0.742 | 0.445 | 0.928 | 0.528 |
|  | Min, Median, Max | 24.6, 48.5, 85.1 | 43.7, 53.2, 69.1 | 39.9, 53.6, 85.4 |  |  |  |  |
| Region 10 | N | 30 | 36 | 30 |  |  |  |  |
|  | Mean (SD) | 49.67 (9.59) | 47.18 (6.21) | 45.95 (9.09) | 0.472 | 0.316 | 0.603 | 0.233 |
|  | Min, Median, Max | 36.9, 48.0, 72.4 | 34.7, 47.0, 57.9 | 24.3, 47.4, 60.2 |  |  |  |  |
| Region 11 | N | 30 | 36 | 30 |  |  |  |  |
|  | Mean (SD) | 42.99 (12.33) | 39.38 (5.90) | 40.78 (7.19) | 0.391 | 0.190 | 0.458 | 0.443 |
|  | Min, Median, Max | 25.0, 42.1, 88.0 | 28.3, 37.9, 54.4 | 24.3, 39.6, 56.1 |  |  |  |  |
| Region 12 | N | 30 | 36 | 30 |  |  |  |  |
|  | Mean (SD) | 37.62 (10.87) | 34.05 (6.39) | 36.25 (5.86) | 0.242 | 0.139 | 0.201 | 0.566 |
|  | Min, Median, Max | 20.9, 37.3, 67.7 | 18.2, 32.9, 55.5 | 26.3, 35.0, 49.7 |  |  |  |  |
|  | | | | | | | | |

*P-values based on test of difference among and between groups using generalized estimating equations (GEE) to account for multiple eyes per subject.
