# Supplementary material for: Evaluation of inner retinal layers as biomarkers in mild cognitive impairment to moderate Alzheimer’s disease
Source: PLoS One. 2018 Feb 8;13(2):e0192646. doi: 10.1371/journal.pone.0192646 (PMC5805310; doi:10.1371/journal.pone.0192646)
Supplement: S6 Table — (DOCX) [file pone.0192646.s007.docx]

| **Variable** | **Statistic** | **Alzheimer** | **Control** | **MCI** | **Overall**  **P-Value*** | **Alzheimer**  **vs Control**  **P-Value*** | **MCI**  **vs Control**  **P-Value*** | **Alzheimer**  **vs MCI**  **P-Value*** |
| --- | --- | --- | --- | --- | --- | --- | --- | --- |
| Region 1 | N | 30 | 36 | 30 |  |  |  |  |
|  | Mean (SD) | 104.6 (20.88) | 94.15 (18.41) | 96.05 (21.55) | 0.241 | 0.096 | 0.760 | 0.230 |
|  | Min, Median, Max | 69.2, 103.6, 158.6 | 61.3, 94.1, 135.9 | 64.3, 95.7, 137.3 |  |  |  |  |
| Region 2 | N | 30 | 36 | 30 |  |  |  |  |
|  | Mean (SD) | 103.1 (23.92) | 99.40 (17.14) | 100.4 (22.28) | 0.854 | 0.574 | 0.875 | 0.725 |
|  | Min, Median, Max | 56.2, 101.8, 165.2 | 72.1, 97.0, 130.7 | 54.7, 101.6, 146.1 |  |  |  |  |
| Region 3 | N | 30 | 36 | 30 |  |  |  |  |
|  | Mean (SD) | 85.80 (26.74) | 78.31 (20.75) | 73.51 (21.39) | 0.320 | 0.337 | 0.478 | 0.131 |
|  | Min, Median, Max | 52.3, 83.4, 184.1 | 46.7, 73.3, 124.8 | 39.1, 71.8, 135.3 |  |  |  |  |
| Region 4 | N | 30 | 36 | 30 |  |  |  |  |
|  | Mean (SD) | 74.65 (23.35) | 65.01 (14.38) | 62.92 (19.87) | 0.231 | 0.132 | 0.680 | 0.098 |
|  | Min, Median, Max | 30.8, 68.8, 132.8 | 41.5, 64.4, 107.6 | 37.4, 58.5, 145.6 |  |  |  |  |
| Region 5 | N | 30 | 36 | 30 |  |  |  |  |
|  | Mean (SD) | 96.35 (33.61) | 87.61 (20.86) | 86.54 (32.45) | 0.543 | 0.298 | 0.889 | 0.328 |
|  | Min, Median, Max | 45.8, 94.5, 182.9 | 54.3, 84.8, 130.0 | 41.7, 82.2, 225.4 |  |  |  |  |
| Region 6 | N | 30 | 36 | 30 |  |  |  |  |
|  | Mean (SD) | 119.0 (38.39) | 118.8 (31.40) | 105.1 (27.97) | 0.287 | 0.986 | 0.156 | 0.213 |
|  | Min, Median, Max | 38.6, 117.1, 221.8 | 65.6, 120.3, 186.7 | 58.8, 100.7, 171.7 |  |  |  |  |
| Region 7 | N | 30 | 36 | 30 |  |  |  |  |
|  | Mean (SD) | 147.3 (35.59) | 147.6 (21.30) | 137.1 (24.91) | 0.288 | 0.971 | 0.121 | 0.286 |
|  | Min, Median, Max | 17.5, 150.7, 208.8 | 114.5, 143.5, 186.1 | 59.4, 139.1, 177.0 |  |  |  |  |
| Region 8 | N | 30 | 36 | 30 |  |  |  |  |
|  | Mean (SD) | 101.4 (34.29) | 102.7 (25.97) | 113.4 (30.09) | 0.423 | 0.886 | 0.241 | 0.255 |
|  | Min, Median, Max | 0.0, 96.3, 172.0 | 63.4, 97.5, 165.3 | 43.4, 119.5, 164.5 |  |  |  |  |
| Region 9 | N | 30 | 36 | 30 |  |  |  |  |
|  | Mean (SD) | 61.07 (17.83) | 59.65 (9.89) | 59.37 (16.82) | 0.932 | 0.733 | 0.947 | 0.742 |
|  | Min, Median, Max | 7.9, 60.7, 91.0 | 42.2, 57.8, 81.6 | 36.4, 57.7, 112.8 |  |  |  |  |
| Region 10 | N | 30 | 36 | 30 |  |  |  |  |
|  | Mean (SD) | 72.69 (14.98) | 71.32 (10.31) | 70.37 (17.47) | 0.895 | 0.721 | 0.831 | 0.652 |
|  | Min, Median, Max | 45.6, 75.2, 101.2 | 51.3, 70.7, 91.3 | 38.2, 67.0, 109.1 |  |  |  |  |
| Region 11 | N | 30 | 36 | 30 |  |  |  |  |
|  | Mean (SD) | 109.3 (28.87) | 110.9 (24.14) | 109.1 (24.12) | 0.964 | 0.843 | 0.802 | 0.980 |
|  | Min, Median, Max | 22.0, 113.8, 164.4 | 65.4, 114.3, 159.6 | 53.7, 106.9, 158.1 |  |  |  |  |
| Region 12 | N | 30 | 36 | 30 |  |  |  |  |
|  | Mean (SD) | 133.3 (27.81) | 130.3 (20.92) | 134.4 (20.53) | 0.800 | 0.680 | 0.516 | 0.872 |
|  | Min, Median, Max | 18.0, 142.2, 170.1 | 86.7, 133.0, 171.4 | 93.8, 136.8, 173.3 |  |  |  |  |

*P-values based on test of difference among and between groups using generalized estimating equations (GEE) to account for multiple eyes per subject.
